# The quest for metabolic biomarkers of IVF outcomes: a meta-analysis and critical review

**DOI:** 10.3389/fcell.2025.1640807

**Published:** 2025-10-17

**Authors:** Pavel Šmak, Jan Juřica, Jana Gregorová, Volodymyr Porokh, Ondřej Peš, Zuzana Holubcová

**Affiliations:** ^1^ Department of Biochemistry, Faculty of Medicine, Masaryk University, Brno, Czechia; ^2^ Department of Pharmacology, Faculty of Medicine, Masaryk University, Brno, Czechia; ^3^ Department of Histology and Embryology, Faculty of Medicine, Masaryk University, Brno, Czechia; ^4^ Reprofit International – Clinic of Reproductive Medicine and Gynecology, Brno, Czechia

**Keywords:** spent culture medium, metabolomics, embryo selection, biomarkers, *in vitro* fertilization, assisted reproduction

## Abstract

Spent culture media (SCM) analysis offers a promising, non-invasive strategy for assessing embryo viability and implantation potential in in vitro fertilization (IVF). By profiling the consumption and secretion of low molecular weight metabolites, SCM analysis may offer valuable insights into embryonic metabolic activity and developmental competence. Identifying reliable biomarkers in SCM has the potential to support more objective embryo selection and reduce time to pregnancy. This Bayesian meta-analysis synthesizes quantitative evidence from studies reporting metabolite concentrations in SCM in relation to IVF outcomes. From a comprehensive literature search identifying 175 studies, 10 met strict inclusion criteria, providing concentration-based data suitable for standardized effect size estimation. Using a multilevel modeling approach, we integrated data across heterogeneous study designs and found seven metabolites positively and ten negatively associated with favorable IVF outcomes. To complement this quantitative synthesis, we critically appraised 14 additional studies excluded from the meta-analysis due to missing calibration data or insufficient methodological transparency. This dual approach highlights recurring methodological challenges and underscores the need for standardized protocols, validated analytical methods, and transparent reporting in SCM research. Overall, the findings illustrate both the potential and the current limitations of SCM metabolic profiling. We provide practical recommendations for improving study design and reproducibility, with the goal of advancing SCM analysis toward clinically relevant biomarker validation.

## Introduction

The selection of viable embryos with the highest implantation potential remains a significant challenge in assisted reproductive technology (ART). Current methods for embryo assessment rely primarily on morphological grading, a subjective approach with limited predictive value (Alpha Scientists in Reproductive Medicine and ESHRE Special Interest Group of Embryology, 2011; Coticchio et al., 2025). Emerging evidence, such as findings from fluorescence lifetime imaging microscopy (FLIM), indicates that metabolic patterns do not consistently correlate with the Gardner criteria widely used for embryo grading in IVF practice (Shah et al., 2022; Venturas et al., 2022). These observations highlight the need for objective, reliable biomarkers capable of predicting an embryo’s potential to implant and lead to a healthy pregnancy.

Embryo development is intricately linked to its microenvironment. *In vivo*, the embryo’s progression toward the implantation site involves dynamic changes in its surroundings and continuous interactions with maternal tissues that support and regulate development (Saint-Dizier et al., 2019; Gualtieri et al., 2024). In contrast, *in vitro* conditions depend on a stationary, low-viscosity culture medium that lacks maternal contributions. In an IVF setting, multiple sibling embryos commonly share the same culture medium, whereas *in vivo*, a single embryo directly interacts with the maternal system, creating a highly individualized developmental environment.

IVF culture media are designed to mimic physiological conditions by maintaining stable pH and osmolarity while supplying essential nutrients to sustain embryo development outside the body (Sciorio and Rinaudo, 2023). *In vitro*, the stationary nature of embryo cultures allows the accumulation of signaling molecules and metabolic byproducts released by the preimplantation embryo (Gualtieri et al., 2024). Analyzing both the nutrients depleted from the medium and the factors secreted by the embryo provides valuable insights into its metabolic activity and developmental potential.

A non-invasive analysis of spent culture media (SCM) composition represents a promising area of research. The fluid in which preimplantation embryos are cultured contains a wealth of biochemical information, including amino acids, lipids, carbohydrates, proteins, non-coding RNAs, cell-free DNA, and extracellular vesicles, making it a valuable resource for assessing embryo viability and implantation potential (Hernández-Vargas et al., 2020; Krisher et al., 2015; Rødgaard et al., 2015; Zmuidinaite et al., 2021). While the role of high-molecular-weight factors as biomarkers of embryo quality has been extensively reviewed elsewhere (Bah et al., 2021; Kanaka et al., 2022; Katz-Jaffe et al., 2009), this article specifically focuses on the low-molecular-weight metabolite composition of SCM and its correlation with IVF outcomes.

Among the various metabolites present in SCM, amino acids (AAs) have been extensively studied for their role in embryo development and their potential as biomarkers of IVF success (Leese et al., 2021). Beyond serving as protein building blocks, AAs contribute to energy metabolism, cellular signaling, and other essential processes. The specific AA requirements of embryos vary depending on developmental stage and environmental conditions. For instance, glutamine is crucial for many cellular functions but can degrade into toxic ammonia in culture media (Yoo et al., 2020). To mitigate this, modern formulations often substitute glutamine with dipeptides such as alanyl-glutamine (Ala-Gln), which provide a more stable source (Song et al., 2025). Other AAs, such as taurine, glycine, and alanine, function as osmolytes, antioxidants, and metabolic precursors (Yancey, 2005). Balancing these components is essential for optimizing embryo development, though their effects may differ across developmental stages, with some studies reporting stimulatory (Gardner and Lane, 1993) or inhibitory (Lane and Gardner, 1994) effects on early cleavage embryos.

Another key component of embryo culture media is the trio of energy substrates - pyruvate, lactate, and glucose (Leese, 2012). Embryonic cells exhibit a distinct energy metabolism, engaging multiple pathways to support growth and epigenetically regulate early differentiation (Milazzotto et al., 2020; Miyazawa and Aulehla, 2018). During the initial cleavage divisions, transcriptional silencing limits biosynthesis, making extracellular pyruvate the primary energy source (Barbehenn et al., 1974). At this stage, amino acids such as glutamine and aspartate also contribute modestly to energy metabolism (Lane and Gardner, 2005).

As preimplantation development progresses, a metabolic shift increases energy demands, leading to enhanced glucose uptake and greater reliance on aerobic glycolysis and oxidative phosphorylation (Lee and Rinaudo, 2024). This phase is also marked by increased lactate production from pyruvate, potentially supporting implantation processes (Gardner, 2015).

The metabolic analysis of SCM holds great potential for IVF; nevertheless, none of the proposed biomarkers have been fully validated, impeding their translation into clinical practice. Previous reviews (Rødgaard et al., 2015; Alizadeh Moghadam Masouleh et al., 2025; Siristatidis et al., 2021; Cimadomo et al., 2024) have provided overviews of SCM studies and pointed to the heterogeneity of study designs, the variability of methodological approaches, and the inconsistency of reported outcomes, thereby underscoring the need for meta-analyses to strengthen the evidence base.

To address this gap, our study integrates the available quantitative evidence on SCM metabolomics and critically evaluates findings in the context of research methodology and data interpretation (PROSPERO registration CRD42025645955). By conducting a meta-analysis, we aim to provide a robust assessment of the relationship between metabolite concentrations and IVF outcomes. Unlike previous reviews, we specifically focus on studies reporting absolute metabolite concentrations, regardless of the analytical platform or clinical endpoint, to enhance the reliability and applicability of our conclusions.

## Materials and methods

### Eligibility criteria

We conducted a systematic literature search using Web of Science in March, 2025, without publication year restrictions. To identify relevant studies, we used the following search terms in the all-fields section: ivf AND spent AND (medium OR media). This simple search strategy aimed to capture maximum studies specifically investigating metabolite concentrations in spent IVF media.

Studies were included in meta-analysis if they met the following criteria: 1) any compound in IVF culture medium was analyzed regardless of the methodology used; 2) the compounds were identified (not only annotated) in regard to specified outcomes; 3) standardized mean difference was given or was calculable; 4) primary data were provided in the text or Supplementary Material. In two cases, primary data were extracted from digitized graph images by manually identifying the vertical pixel position of each data point on significantly enlarged images. These pixel positions were converted to scientific units using a linear calibration derived from the graph’s vertical axis scale. Studies were excluded from the meta-analysis if they: 1) were not human; 2) focused on pattern comparison; 3) used ratios instead of effect size; 4) provided only signal responses (e.g., peak intensities or areas) instead of concentration levels obtained by means of calibration of the used detector signals.

### Data extraction and analysis

Due to the limited number of studies within each subgroup, the specified outcomes (e.g., clinical pregnancy, blastocyst formation, euploidy) were pooled across all outcomes. In the original publications, the effect size (standardized mean difference) for each metabolite was calculated as the difference in means between the endpoint success and endpoint failure groups. This difference was then standardized by dividing it by the pooled standard deviation (SD_pooled_) of both groups, calculated as:

$${SD}_{pooled}=\sqrt{\frac{\left(n_{1}-1\right){\left({SD}_{1}\right)}^{2}+\left(n_{2}-1\right){\left({SD}_{2}\right)}^{2}}{n_{1}+n_{2}-2}}$$

where n_1_ and n_2_ are the sample sizes, and SD_1_ and SD_2_ are the standard deviations of the success and failure groups, respectively. When studies reported standard errors (SE), these were converted to standard deviations using the formula:

$$\mathrm{SD}=\mathrm{SE}\times \sqrt{n}$$

The meta-analysis was performed under a Bayesian paradigm using R software (Team, 2022) with packages brms (Bürkner, 2017), tidyverse (Wickham et al., 2019), and tidybayes (Kay, 2024). As all included studies provided complete data for the metabolites of interest, no imputation methods were required for handling missing data. Sparsity in the cross-design was handled by employment a full probabilistic model as follows:

$$\begin{array}{l} \;\text{Outcome }(\text{Likelihood}):\\ \;SMD_{i}∼\text{Normal}\left(\mu_{i},\sigma \right)\\ \;\mu_{i}=\beta_{0}+\beta_{m\left[i\right]}+u_{0j\left[i\right]}+u_{m\left[i\right]j\left[i\right]}\\ \;\text{Study}-\text{Level Effects }(\text{Hierarchical}):\\ \;\left[\begin{array}{c} u_{0j}\\ u_{1j}\\ \vdots \\ u_{25j} \end{array}\right]∼\text{MVNormal}\left(0,\Sigma_{u}\right)\\ \;\Sigma_{u}=\text{diag}\left(\tau \right)\cdot R\cdot \text{diag}\left(\tau \right)\\ \;\text{Priors}:\\ \;\beta_{0}∼\text{Normal}\left(0,5\right)\\ \;\beta_{m}∼\text{Normal}\left(0,2\right)\\ \;\tau ∼\text{Exponential}\left(0.1\right)\\ \;R∼\text{LKJ}\left(4\right)\\ \;\sigma ∼\text{Exponential}\left(1\right) \end{array}$$

The index (i) denotes individual publications that reported concentrations of the metabolite (m). The parameters (β_0_, β_m_) represent global intercept and metabolite offsets, which were treated as dependent and thus modeled jointly using a covariance matrix (Σ), which has been decomposed to expose a correlation (R) matrix. The pooled effect size standard deviation diagonal matrix (*τ*) represents the variation of the pooled effect sizes across studies and metabolites. It is worth noting that such a complex model would not converge using classical statistics; however, using the full probabilistic approach and weakly informative priors it is completely viable to address the study and metabolite heterogeneity without compromising the performance and/or predictive accuracy. A maximum compatibility model was applied to strike a balance between flexibility and regularization, helping prevent overfitting in sparse datasets (Barr et al., 2013).

## Results

### Study selection

**FIGURE 1:**
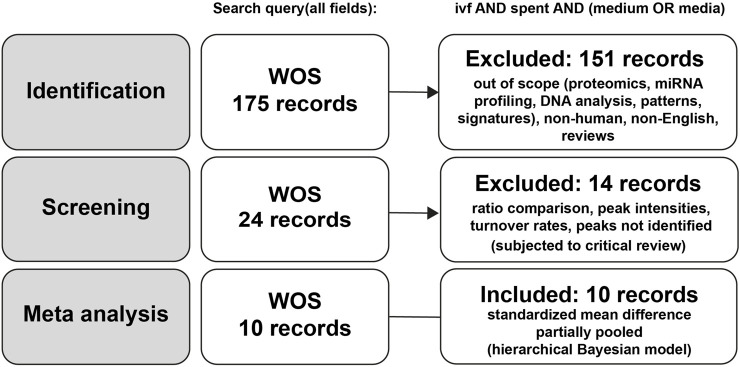
Flowchart of study selection for meta-analysis.

A systematic literature search in the Web of Science database initially identified 175 records (Supplementary Table S1). After the identification phase, 151 studies were excluded for being out of scope, including those focused on proteomics, miRNA, profiling, DNA patterns, metabolic signatures, non-human studies, non-English publications, and reviews. Following this initial screening, 24 records remained eligible for further assessment. A second screening phase led to the exclusion of 14 additional studies due to reliance on ratio comparisons, unidentified metabolites, peak intensities without quantitative data, or turnover rate analyses, which did not meet the inclusion criteria for meta-analysis (Figure 1). Although excluded from the meta-analysis, these studies were subjected to the critical review to evaluate their methodological design, limitations, and contribution to the field. Ultimately, 10 studies were included in the meta-analysis, yet they remained heterogeneous in culture conditions, clinical endpoints, and sampling time (Table 1). Furthermore, the selected SCM studies employed various analytical approaches with differing sensitivities. Some of them focused on a single metabolite (Boyama et al., 2016; Ferrick et al., 2020; Gardner et al., 2011; Li et al., 2013; Miao et al., 2022), while others assessed multiple analytes (Gardner et al., 2001; Huo et al., 2020; Motiei et al., 2020; Nami et al., 2024; Olcay et al., 2022).

**TABLE 1:** The studies included in meta-analysis.

#	Clinical endpoint	Sampling day	Medium analyzed	(n) samples (+/−)	Culture type	O_2_ (%)	Control	Analytical method	Metabolite analyzed	Study
1	Blastocyst formation	4; 5; 6	G2.2 (modified in-house)	25/18	Individual	5	No	UMF	Glc, Pyr	(Gardner et al., 2001)
2	Clinical pregnancy (fetal heartbeat)	4; 5	G2 (Vitrolife)	29/21	Individual	5	Yes^a^	microfluorimetry	Glc	(Gardner et al., 2011)
3	Clinical pregnancy (fetal heartbeat)	3	G1 (Vitrolife)	52/50	Individual	n.a	Yes^a^	SPM	NH3	(Li et al., 2013)
4	Clinical pregnancy (gestational sac)	3	HTF (Lonza)	16/24	Individual	20	Yes	Enzymatic Assay Kit	Hcy	(Boyama et al., 2016)
5	Clinical pregnancy (gestational sac)	3	n.a	52/46	Group	n.a	Yes	HPLC	Asp, Ser, Gly, His, Tau, Arg, Thr, Ala, Pro	(Huo et al., 2020)
6	Clinical pregnancy (fetal heartbeat)	5	G-TL (Vitrolife)	17/20	Individual	5	Yes	UMF + LC-MS	Glc	(Ferrick et al., 2020)
7	Blastocyst formation	5	G-TL (Vitrolife)	n.a. (58 cycles)	Individual	n.a	Yes	CE	His, Ile, Leu, Thr, Val, Trp, Phe, Met, Arg, Ala, Asp, Glu, Gly, Asn, Gln, Ser, Tyr, Tau, Ala-Gln, Glc, Pyr, Lac	(Motiei et al., 2020)
8	Blastocysts PGT (euploid/aneuploid)	3	G1 (Vitrolife)	30/30	Individual	5	Yes^a^	GC-MS	Gln	(Miao et al., 2022)
9	Blastocysts PGT (euploid/aneuploid)	5/6	CSCM-C (Irvine Scientific)	40/71	Individual	n.a	Yes^a^	LC-MS/MS	Ala, Arg, Gly, His, Pro, Ser, Thr, Tyr	(Olcay et al., 2022)
10	n.a. (RIF patients/healthy controls)	3	SAGE 1-Step (Origio)	15/35	Group	5	No	SPM + HPLC	Pyr, Glc, Lac, His, Met, Phe, Glu, Asp	(Nami et al., 2024)

n. a., not available in the original publication, PGT, preimplantation genetic testing; RIF, recurrent implantation failure, Hcy = Homocysteine, Tau = Taurine, Glc = Glucose, Pyr = Pyruvate, Lac = Lactate, UMF, ultramicrofluorescence; SPM, spectrophotometry; HPLC, High-performance liquid chromatography; CE, capillary electrophoresis; GC-MS, Gas chromatography–mass spectrometry; LC-MS/MS, liquid chromatography with tandem mass spectrometry.

^a^ The inclusion of control samples is stated, but how they were used for data correction or normalization was not specified.

### Meta-analysis

**FIGURE 2:**
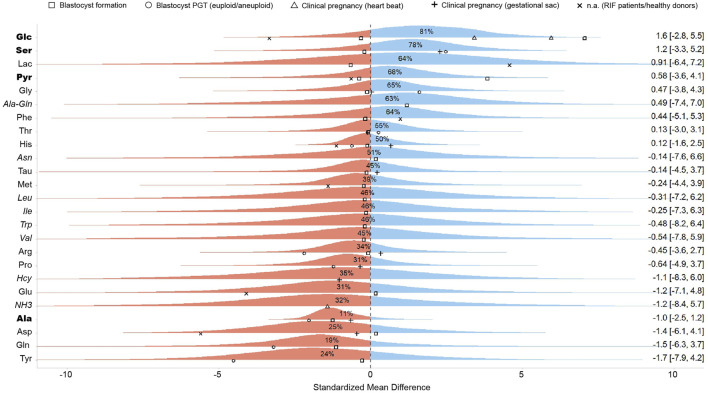
Pooled standardized mean differences of metabolites across all studies. This figure presents the aggregated effect sizes, where the right-hand side of the plot displays the mean and a 95% credibility interval for each metabolite. Points represent the standardized mean difference of individual studies, with different shapes denoting the specific clinical outcome investigated. The percentage values indicate the probability that the pooled effect size for the given metabolite is greater than zero, summarizing the overall trends across studies. Blue and red color represents positive and negative effects, respectively. Metabolites reported by a single study are shown in italics and should be regarded as low-confidence evidence. Metabolites supported by three or more outcome reports with a consistent directional trend are shown in bold, highlighting the most reproducible signals in the dataset.

**FIGURE 3:**
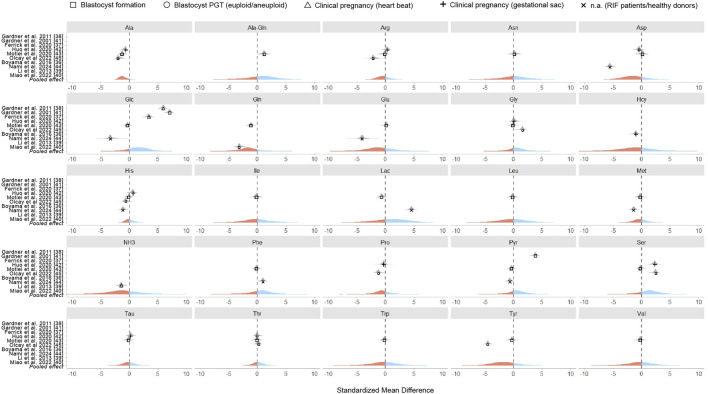
Standardized mean differences of metabolites in individual studies. Each panel displays the results for a specific metabolite, with points representing a standardized mean difference of individual studies. Point shapes indicate the clinical outcome assessed while the pooled effect size across all studies is visually represented by color - with blue indicating positive effects and red indicating negative effects.

The meta-analysis was designed as a Bayesian multilevel model of the standardized mean difference across all studies reporting metabolite concentrations, with outcomes classified as successful or unsuccessful based on specific clinical endpoints. Figure 2 presents the pooled effect sizes for each metabolite, while Figure 3 shows study-specific results, highlighting variation across datasets. It is worth noting that a classical meta-analysis would not have detected significant pooled effects, as all metabolite estimates include zero within their variation. In the current research, an effect size is considered statistically significant when its estimate lies at least two standard errors from zero.

Our meta-analysis found an association between embryo viability and the abundance of seven metabolites in SCM, with a probability of effect (Pr (*μ* > 0)) of at least 0.60 (Figure 2). These included three carbohydrates (lactate [Lac], glucose [Glc], and pyruvate [Pyr]), three AAs (phenylalanine [Phe], glycine [Gly], and serine [Ser]), and one dipeptide (alanine-glutamine [Ala-Gln]). Since these metabolites are routinely supplemented in culture media, their increased presence in SCM could reflect lower uptake by high-quality embryos. In contrast, ten metabolites (Gln, Ala, Tyr, Asp, Glu, NH_3_, Pro, Hcy, Arg, Met) had negative pooled effect sizes (Pr (*μ* > 0) < 0.40), indicating an association with poor embryo quality (Figure 2). Notably, Ala (Pr (*μ* > 0) = 0.07) and glutamine (Pr (*μ* > 0) = 0.20) exhibited strong negative associations with embryo viability. However, these associations should be interpreted with caution given the limited evidence base and variability across studies.

The wide credible intervals observed in our analysis underscore the imprecision of the estimates, which is a direct consequence of the limited sample sizes and substantial between-study heterogeneity. For several metabolites only a single study reported concentration data, resulting in very uncertain effect estimates. We included these in the pooled analysis to provide a comprehensive overview of all metabolites for which quantitative data exist, but we stress that such single-study findings should be regarded as exploratory only. Accordingly, in Figure 2 these metabolites are marked as low-confidence evidence. By contrast, metabolites supported by multiple outcome reports with a consistent directional trend offer more consistent evidence and may represent more promising candidates for further validation.

### Critical review of SCM studies lacking primary data

Special consideration is needed for studies that report 1) metabolite ratios, 2) peak intensities or areas, or 3) turnover rates instead of standardized mean differences. Even if raw data are presented, reanalyzing them using a different statistical approach than originally intended is not always feasible. As a result, these studies could not be directly included in the meta-analysis. The following subsections critically examine the key methodological challenges observed in excluded studies.

#### Analyte ratio evaluation

Several studies reported outcome ratios or analyte ratios instead of standardized mean differences, often focusing on contrasts linked to opposing outcomes (Brison et al., 2004; Seli et al., 2008; Zhao et al., 2013; Wallace et al., 2014; Mádr et al., 2015; Zivi et al., 2014). However, the rationale for selecting these ratios and whether appropriate statistical adjustments for multiple comparisons were applied is often unclear. Although some authors justified the use of ratios as a means to reduce variability, this approach should be applied with caution. Ratio-based analyses can introduce bias, especially when the normal distribution is assumed without proper validation.

#### Peak intensities or areas

Our meta-analysis excluded studies that did not clearly define the relationship between analyte concentration and response. If only detector responses such as peak intensities or areas were provided, these might have served for relative comparisons within the original publication; however, the results are not suitable for any later quantitative synthesis. Examples of such studies include those analyzing metabolomic signatures using MALDI MS (Pais et al., 2020), NMR (Pudakalakatti et al., 2013; D'Souza et al., 2016), and GC MS (Deng et al., 2024).

#### Turnover rates

A more advanced approach to metabolite analysis involves measuring analyte concentrations over multiple days of incubation and comparing outcomes within a time series framework. However, accurately determining turnover rates presents significant challenges. AA levels detected in the SCM can be influenced by multiple factors, including blank media composition and culture conditions (Drábková et al., 2017), sampling time (Skrutková Langmajerová et al., 2022), and recovery dynamics in cryopreserved embryos (Stokes et al., 2007). Variability in these factors across studies complicates direct comparisons and may affect the reliability of turnover rate assessments. Nevertheless, this approach could gain value when combined with microfluidic technologies, which provide a dynamic and controlled culture environment that better mimics *in vivo* conditions and may help standardize experimental settings across studies.

## Discussion

This critical review assesses the potential of metabolic substrates in SCM as biomarkers of embryo quality. Despite the growing interest in non-invasive embryo assessment, translating SCM analysis into clinical practice remains challenging due to inconsistencies across published studies. Only ten studies met the criteria for meta-analysis, underscoring the lack of standardization in study design and data reporting.

Key disparities limiting research result comparability and the identification of reliable biomarkers include 1) variations in culture conditions, 2) inconsistencies in outcome definitions, and 3) shortcomings in reporting practices. Standardizing methodologies and improving study designs are crucial steps toward fully realizing the potential of SCM analysis in IVF.

### Culture conditions

Most IVF clinics rely on commercially available, ready-to-use culture media, which offer greater consistency than historically used in-house formulations. However, their full composition is often undisclosed due to proprietary restrictions. Studies examining the composition of IVF media have identified discrepancies between manufacturers, underscoring a lack of standardization in clinical embryo culture practices (Zagers et al., 2025; Morbeck et al., 2014).

Nutrient availability and differences in oxygen concentration during culture can significantly impact embryo metabolism and, consequently, the SCM composition (Konstantogianni et al., 2024). Given its critical role in embryo metabolism, oxygen concentration should be consistently documented in the literature. Notably, even control media cultured without embryos can change over time due to substrate degradation at 37 °C and exposure to oxygen tension. Without appropriate controls to account for these alterations, comparisons between metabolomic data from successful and unsuccessful cycles risk oversimplification and potential misinterpretation.

Additionally, the type of embryo culture further complicates the interpretation of SCM studies and limits the comparability of results. In group culture, multiple embryos share and modify the environment. Factors such as the total number of embryos and the ratio of high- to low-quality embryos influence paracrine signaling, thereby altering SCM composition (Tao et al., 2013). These collective metabolic interactions can obscure embryo-specific signals, making it difficult to attribute observed changes to individual embryos. In contrast, individual culture offers a more controlled setting, allowing clearer identification of metabolic “fingerprints” directly associated with a single embryo.

Beyond culture conditions, other ART specific factors such as maternal age and the use of cryopreserved oocytes or embryos are also likely to influence embryonic metabolic status and, consequently, SCM profiles. Evidence from humans and animal models demonstrates their impact on embryonic metabolism (Stokes et al., 2007; Khurana and Niemann, 2000; Balaban et al., 2008; Kurzella et al., 2024; Trohl et al., 2023; Hashimoto and Morimoto, 2022), underscoring the importance of including these variables in future SCM analyses in the IVF setting and, where possible, stratifying results accordingly.

Ultimately, standardizing culture conditions, incorporating robust controls and accounting for confounding factors are essential to establish reliable baseline values, distinguish embryo-specific metabolic signatures, and ensure the validity of SCM analysis.

### Endpoint definitions

A major limitation in the interpretation and comparison of SCM studies is the inconsistency in endpoint definitions. Terms such as “good/bad quality”, “(non-)developing”, “(non-) blastula” or “growing/impaired embryos” lack standardized criteria, making it difficult to replicate findings or assess the predictive value of specific metabolites (Miao et al., 2022; Motiei et al., 2020; Zheng et al., 2021). Additionally, definitions of clinical pregnancy vary among authors; some consider the detection of a fetal heartbeat as the endpoint (Ferrick et al., 2020; Gardner et al., 2011; Li et al., 2013), while others rely on the presence of a gestational sac (Boyama et al., 2016; Huo et al., 2020). Notably, none of the studies included in the meta-analysis used live birth as an outcome, despite its status as the most clinically relevant measure of IVF success.

To improve comparability and reproducibility, standardized criteria for embryo quality assessment should be consistently applied. The Gardner grading system, which evaluates blastocyst morphology, remains the most widely used approach in clinical practice. However, morphology-based assessment is inherently subjective and prone to inter-observer variability. In contrast, preimplantation genetic testing (PGT) provides an objective molecular assessment of embryo chromosomal status and therefore represents a more reproducible benchmark for correlating metabolic profiles with embryo quality. In parallel, clearly defined pregnancy markers (such as β-hCG levels, gestational sac detection, and fetal heartbeat confirmation) are essential to ensure consistency in outcome reporting.

The association of SCM profiles with individual embryo viability becomes particularly problematic in the context of transferring multiple embryos. When a singleton pregnancy results from the transfer of two embryos, it is impossible to determine which embryo implanted, introducing ambiguity that undermines efforts to correlate specific SCM profiles with implantation success (Li et al., 2013; Huo et al., 2020). This uncertainty limits the precision of outcome attribution and impedes the validation of metabolic biomarkers. In contrast, transferring a single embryo removes this confounding factor, allowing any resulting singleton gestation to be directly linked to the metabolic profile of that embryo. Therefore, studies aiming to establish robust SCM-based biomarkers would benefit from prioritizing single embryo transfers to achieve more reliable and interpretable outcomes.

### Sampling challenge and analytical methodology

The sampling procedure is crucial for obtaining valid and comparable results in SCM analysis. A key concern is whether the collected sample accurately represents the embryo’s microenvironment, as metabolic gradients can create heterogeneity in SCM. This is particularly important in group culture, where sampling position may lead to over- or underrepresentation of certain signals. Proper homogenization during and after embryo removal enhances sample representativeness and accuracy.

In individual embryo cultures, collecting samples from small droplet volumes under oil is inherently difficult, posing a technical challenge. Furthermore, the limited sample size may fall below the sensitivity thresholds of many analytical methods, affecting the detection and quantification of certain metabolites. Given the complexity of embryo metabolism, comprehensive metabolic profiling is more likely to provide meaningful insights than single-metabolite assessments.

The complexity of metabolomic data necessitates advanced statistical approaches for both targeted and untargeted analyses. However, the small sizes of SCM samples limit statistical power. The common practice of reporting only ratios and peak intensities, without calibrating to the concentration levels of observed metabolites, also reduces reproducibility and compromises result interpretation. Furthermore, a focus on statistical significance over biological relevance increases the risk of misleading conclusions.

Another major issue is inconsistency in sampling time points, as researchers analyze SCM collected at different stages of embryo development, ranging from day 3 to day 6. Some studies collect a single sample (either at the end of cultivation (Boyama et al., 2016; Li et al., 2013; Huo et al., 2020; Motiei et al., 2020; Olcay et al., 2022) or at an intermediate stage (Ferrick et al., 2020; Miao et al., 2022), while others perform time-course sampling at multiple points (Gardner et al., 2011; Gardner et al., 2001). This variability complicates cross-study validation and underscores the need for standardized sampling protocols to improve reproducibility and enhance data validity across studies.

Since embryonic nutrient requirements shift at compaction, the timing of sample collection directly influences metabolite concentrations detected in SCM. Cross-study pooling should therefore take into account that metabolic activity differs substantially between cleavage-stage and blastocyst-stage embryos. It is SCM analysis within the same developmental stage that may help identify genetically abnormal or otherwise stressed embryos through altered metabolic patterns. Sampling on day 3 can provide insight into cleavage-stage physiology, whereas day 5–6 sampling is likely more informative for predicting implantation potential in clinical IVF.

### Correlation vs. causation: a persistent challenge

A fundamental question in SCM analysis is whether the detectable metabolite concentrations are a cause or a consequence of embryo development. Embryos with higher developmental potential may metabolize available nutrients differently from those with compromised viability. This bidirectional interaction highlights the risk of conflating correlation with causation. The field must remain cautious about drawing premature conclusions regarding causality, emphasizing the need for a deeper understanding of the complex relationships between embryo metabolism, SCM composition, and IVF outcomes.

In this context, the potential impact of “survivorship bias” should not be overlooked. The lack of published results reporting no association between SCM analysis and IVF outcomes creates an imbalance in the literature, making it difficult to determine whether all reported findings genuinely reflect causative relationships or are merely artifacts of selective reporting (publication bias). This inherent bias, where studies with nonsignificant results are less likely to be submitted or accepted for publication, distorts our understanding of the true impact of SCM analysis.

### The path forward

**FIGURE 4:**
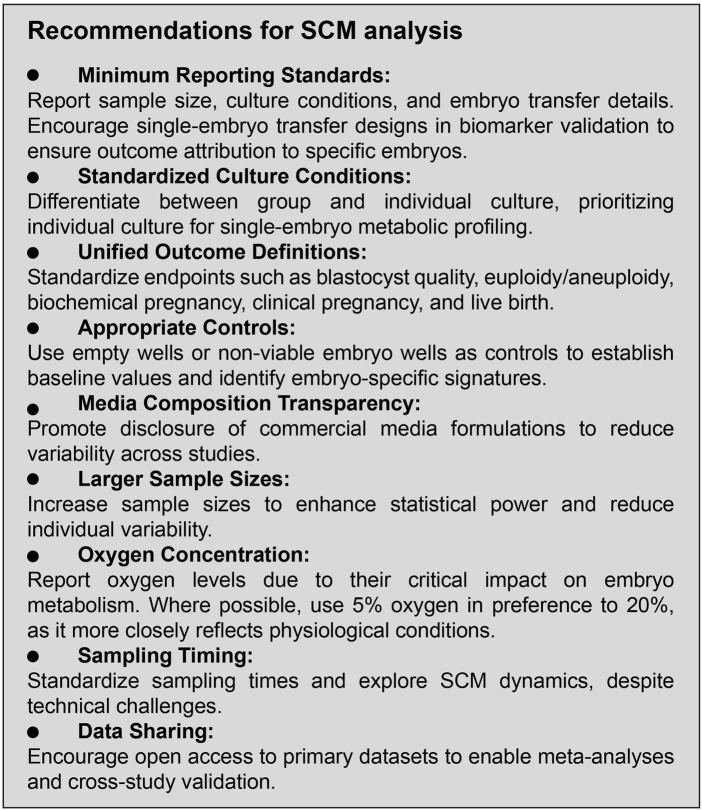
Study design recommendations.

To advance the utility of SCM analysis, the field must emphasize the development of standardized protocols for study design, culture conditions, sampling procedure, endpoint definitions, and transparent reporting. Well-designed, large-scale studies with clearly defined criteria and robust controls are essential for generating high-quality evidence. Figure 4 summarizes our recommended study design guidelines to enhance methodological rigor and reproducibility in SCM analysis.

Additionally, basic research should focus on deepening our understanding of the dynamic interactions between embryos and their microenvironment, considering both intrinsic and extrinsic factors that influence metabolic activity. Integrating SCM profiling with emerging technologies, such as artificial intelligence (AI) and multi-omics approaches (Siristatidis et al., 2021; Bori et al., 2021), may further refine predictive models and pave the way for more personalized and effective IVF strategies. Recent work by Cabello-Pinedo et al. (2024) (Cabello-Pinedo et al., 2024) exemplifies this direction, showing that untargeted metabolomics combined with pathway enrichment can pinpoint potential biomarkers for SCM profiling, while the mathematical integration of metabolite concentrations using AI-based algorithms predicted implantation outcomes with an accuracy of around 85%. This underscores artificial intelligence as the most promising tool currently available for translating complex metabolomic data into clinically meaningful predictions.

Looking ahead, combining SCM metabolomics with time-lapse morphokinetic assessment could capture complementary aspects of embryo development. While time-lapse imaging offers detailed information on developmental dynamics, SCM metabolomics provides molecular insight into the biochemical processes occurring within the embryo. At present, neither method alone provides sufficient predictive accuracy to support clinical decision-making. The integration of these approaches, particularly when combined with AI-based analysis, may ultimately yield more precise and clinically useful embryo selection tools.

## Conclusion

SCM analysis presents a promising non-invasive approach for assessing embryo viability and implantation potential in IVF. In this review, we conducted a Bayesian meta-analysis of the available literature and identified seven metabolites associated with positive outcomes and ten with negative outcomes. While these findings highlight the potential of metabolic profiling to support embryo selection, they must be regarded as exploratory. At present, there is insufficient evidence to support clinical application, as the conclusions are constrained by substantial heterogeneity in study design, outcome definitions, and analytical methods.

Technological advances in molecular profiling can reveal additional biomarkers that could enhance embryo selection beyond traditional morphological assessment. However, before any clinical translation is possible, the field must first establish standardized protocols for study design, culture conditions, sample collection, and data reporting. Enhancing reproducibility and cross-study comparability is essential for validating clinically relevant SCM-based biomarkers. Achieving this goal will require a concerted effort from researchers, clinicians, and industry stakeholders.

Future research should prioritize large-scale, well-controlled studies with clearly defined endpoints to assess the clinical utility of candidate biomarkers in SCM. Adherence to best research practices and the integration of emerging technologies will be critical to fully realizing the potential of SCM analysis in improving embryo selection and IVF outcomes. Until such validation is achieved, SCM analysis should remain an investigative research tool rather than a clinical decision-making aid.
